# Sexual and reproductive health knowledge among adolescent Syrian refugee girls displaced in Lebanon: The role of schooling and parental communication

**DOI:** 10.1371/journal.pgph.0001437

**Published:** 2023-01-09

**Authors:** Sasha Abdallah Fahme, L’Emira Lama El Ayoubi, Jocelyn DeJong, Maia Sieverding

**Affiliations:** 1 Department of Health Promotion and Community Health, Faculty of Health Sciences, American University of Beirut, Beirut, Lebanon; 2 Department of Epidemiology and Population Health, Faculty of Health Sciences, American University of Beirut, Beirut, Lebanon; University of Toronto, CANADA

## Abstract

Adolescent Syrian refugee girls living in Lebanon are vulnerable to poor sexual and reproductive health (SRH). Sociocultural norms, stigmatization, and limited educational opportunities in the context of forced displacement may impact adolescent girls’ SRH. Little is known about how and where girls in this population access SRH information and services. This study aimed to: (1) assess knowledge of SRH topics among a population of adolescent Syrian refugee girls displaced in Lebanon, and (2) determine the association of schooling versus maternal SRH communication with SRH knowledge. A total of 418 11-17-year-old Syrian refugee girls displaced in the Beqaa region of Lebanon were recruited to participate in a cross-sectional survey. Bivariate logistic regression and ordinary least squares regression models were used to examine the associations between schooling, maternal SRH communication, and other covariates with SRH knowledge outcomes. Significant predictors (p<0.2) were included in multivariate analyses. The mean age of girls was 13.4 years. Approximately two thirds of our sample was enrolled in school, with enrollment rates dropping considerably around age 15. In bivariate and multivariate models, older age and participation in SRH programs were predictive of puberty knowledge. One in five girls enrolled in school had learned about menstruation in school, which was associated with higher puberty knowledge in bivariate models. Older age, current school enrollment, and reaching the 8^th^-11^th^ grade were strongly associated with HIV knowledge. Schooling is more strongly associated with SRH knowledge among adolescent girls than is maternal communication. School-based SRH curricula should be administered on the basis of age and not grade, given significant age-for-grade heterogeneity in this population. Forced displacement and poverty are major barriers to education retention and may have long-term impacts on girls’ health.

## Introduction

Conflict-affected and forcibly displaced adolescents in low- and middle-income countries (LMICs) represent a vulnerable population at risk of adverse sexual and reproductive health (SRH) outcomes related to challenges of forced migration, including exploitation, early marriage and gender-based violence [1–3]. Despite these risks, a growing body of literature suggests that adolescents in displacement settings have low knowledge of and access to SRH services [3]. These disparities may be further exacerbated in patriarchal societies with disempowering gender norms, such as those in the Middle East and North Africa (MENA) [4], a region, in which adolescent SRH may not be prioritized or altogether neglected [5–7].

Adolescent Syrian refugee girls displaced in Lebanon are one such population thought to be at high risk of poor SRH outcomes, such as reproductive tract infections and adolescent pregnancy [8, 9]. There are an estimated one to 1.5 million Syrian refugees displaced in Lebanon, half of whom are female and over half of whom are under 18 years of age, representing the highest per capita refugee population in the world [10]. The overwhelming majority of Syrian refugees in Lebanon live in extreme poverty and gendered obstacles to education and employment heavily restrict opportunities for social mobility [10–13]. In 2021, only 13% of Syrian adolescents were enrolled in school, with marriage most commonly cited among girls, but not boys, as the primary reason for non-attendance [10]. In this context, previous studies have shown that access to accurate SRH information in this population is limited by low school enrollment rates, weaknesses in the healthcare system, and sociocultural norms that stigmatize adolescent sexuality, particularly among unmarried adolescents [8, 14, 15].

**Fig 1:**
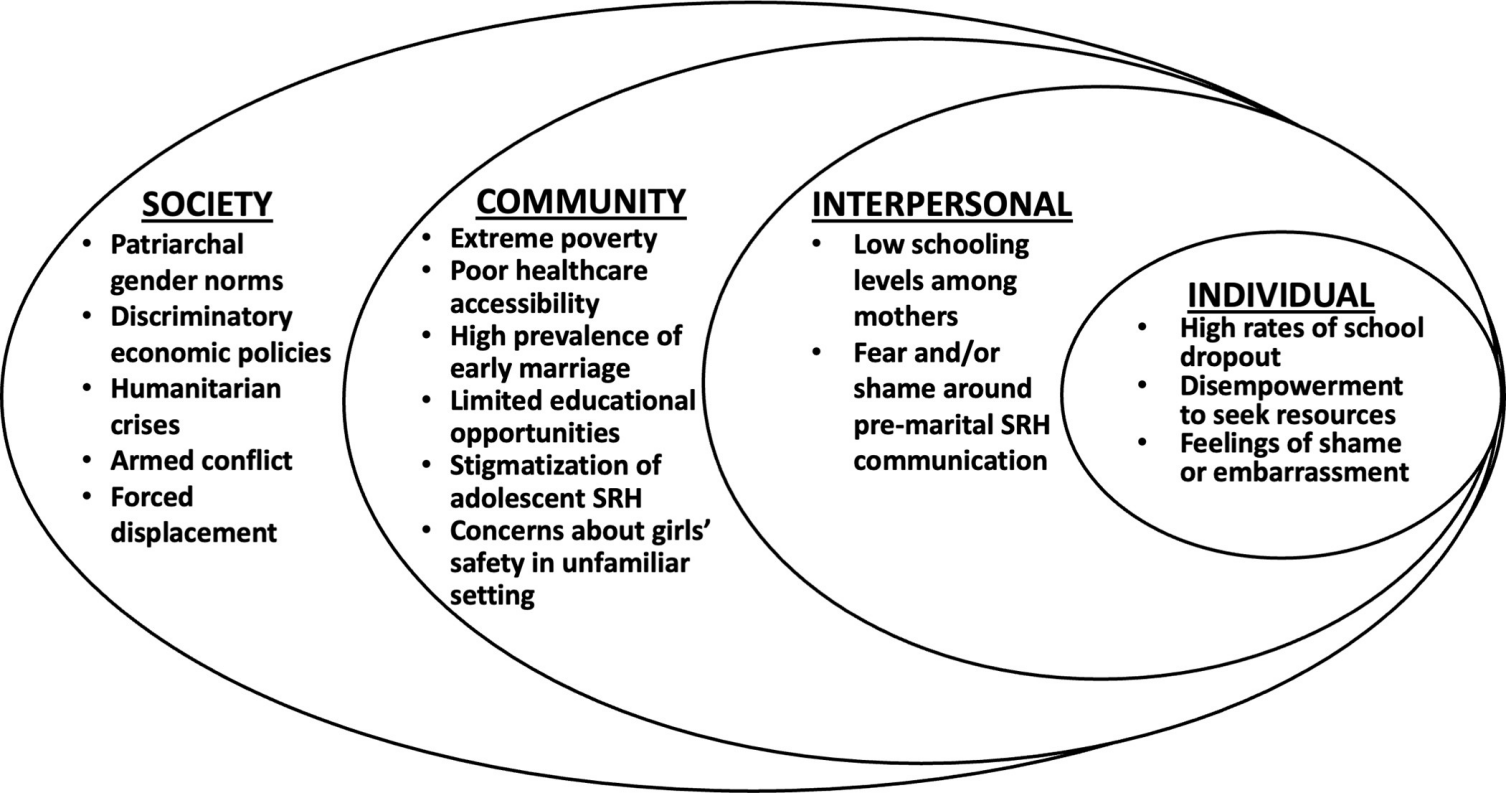
Social ecological framework for barriers to accessing SRH information among adolescent Syrian refugee girls.

Barriers to accessing SRH information and resources among adolescent Syrian refugee girls in Lebanon may be illustrated using a modified social ecological framework [16], which incorporates both displacement-driven factors and stigmatizing sociocultural norms that may disproportionately impact adolescent girls (**Fig 1**). While disruptions in education due to armed conflict, forced displacement and extreme poverty may be associated with poor SRH outcomes among adolescent Syrian girls in Lebanon [8], the individual impact of the determinants outlined in the framework on adolescent health in this context has not been studied and remains unknown. At the community-level, stigmatizing cultural norms limit the integration of comprehensive sexuality education (CSE) curricula into the Lebanese educational system [14], such that school-based SRH curricula for both Syrian and Lebanese students are often incomplete and/or taught in the context of human biology, without mention of contraception or sexually transmitted infections (STIs) [17].

Syrian adolescents may experience additional barriers to accessing SRH information, which are specific to the context of forced displacement. It is common for Syrian adolescents who are in school to be in grades lower than that expected for their age due to displacement-, poverty- and learning-related barriers [18, 19]. Syrian adolescents are thereby at risk of dropping out of school prior to reaching a grade in which SRH information is taught. Additionally, Syrian students in Lebanon typically attend condensed, afternoon sessions at public schools [20], which may forego SRH curricula due to prioritization of other subjects. This represents a missed opportunity to convey SRH information, as globally schools have been demonstrated to be a convenient and cost-effective setting for age-appropriate CSE [17, 21] because adolescents can be reached at scale through these existing institutions. There is overwhelming evidence from diverse settings that CSE improves SRH knowledge, fosters positive attitudes, and empowers adolescents to make informed decisions and communicate openly with their parents [17]. Further, educators are typically trusted by both adolescents and parents to provide accurate information in an accessible manner [17].

Given these limitations in CSE accessibility, parent-child communication may be a significant source of SRH information for adolescent Syrian refugees in Lebanon, and particularly those who are not enrolled in school. Mothers specifically have been found to be the most important source of information on menstruation for girls living in multiple LMICs [22, 23]. However, cultural taboos and fear that communication might encourage risky sexual behavior have been identified as barriers to effective parent-child SRH communication in LMICs, including countries in MENA [24, 25]. Indeed, reflective of conservative gender norms in Arab societies, multiple studies have shown that, among Syrian mothers, shame is a major barrier to maternal SRH communication, given widespread reluctance to transmit information about SRH to unmarried girls [14, 25]. Syrian mothers may prefer their daughters to receive information from other sources including female relatives and religious authorities [14, 25]. Additionally, there are several issues related to the accuracy of information provided by parents, who themselves are unlikely to have received formal SRH education, and may unknowingly pass along misinformation to their children [1, 22], or feel under-qualified to lead such discussions [4].

While interventions to improve the SRH of adolescent Syrian refugee girls should be comprehensive and address multiple health determinants [8], there is limited data on where and how adolescent Syrian refugee girls in Lebanon access SRH information and services. There have similarly been no studies rigorously examining the correlates of SRH knowledge in this population. This paper aims to fill this gap by: 1) examining adolescent Syrian refugee girls’ knowledge of SRH topics, including menstruation, puberty, STIs, and contraception, and 2) testing the hypothesis that schooling, rather than maternal-child communication, is more strongly associated with accurate SRH knowledge in this population.

## Methods

### Study design and setting

The survey upon which our analysis is based was conducted as part of the *Amenah* project, a multi-component, community-based intervention to mitigate the drivers of early marriage and improve SRH among adolescent Syrian refugee girls in the Beqaa governorate of Lebanon. The intervention was piloted in 2018 and an expansion phase planned for 2019–2020. The survey we use, conducted between December 2019 and March 2020, was intended to be the baseline for the expanded intervention. However, data collection was suspended and the intervention postponed in mid-March 2020 due to the COVID-19 pandemic. We therefore analyze the data as a cross-sectional survey of adolescent Syrian refugee girls in this semi-rural region, which borders Syria to the east and hosts the highest number of Syrian refugees in the country [26]. There are no formal Syrian refugee camps in Lebanon [27]; Syrian refugees residing in the study area live either within the host (Lebanese) community, predominantly in rented apartments, or in informal tented settlements (ITS). ITS in the study area vary considerably in size; some consist of 10–20 households whereas others contain several hundred people. Participants in the study were recruited from approximately 30 different ITS as well as non-camp areas.

### Ethics statement

The study was reviewed and approved by the Institutional Review Board of the American University of Beirut. Written consent for each girl to participate was obtained from both of the girls’ parents or her legal guardian(s). Following parental consent, participants provided oral assent prior to completing the survey.

### Eligibility and recruitment

The study was open to all Syrian girls aged 11–17 as of January 1, 2020 residing in the intervention catchment area, regardless of schooling or marital status. Our target sample size for the evaluation of the intervention was 625 girls. Recruitment was conducted through multiple mechanisms. To recruit girls who were out-of-school or attending informal schools, female Syrian community workers (CWs) recruited from the community to work with the study went door-to-door in ITS. In-school girls were recruited through local public and private schools. Families of girls who participated in the 2018 pilot of the intervention were re-contacted by phone and invited to participate in the expanded intervention. At the time that recruitment was halted due to the COVID-19 pandemic, a total of 810 girls had been recruited as potentially eligible for and interested in participation, including 163 girls from the pilot and 647 girls newly registered by their parents.

Following recruitment, each household was assigned to a CW to conduct parental consent and complete a household survey. Of the girls whose households had been approached for consent prior to the study being halted, 29 (4%) were determined to be ineligible due to age or being outside the geographic area of the study. Another 11 girls could not be recontacted. The refusal rate at the point of consent was 25% (199 girls), in most cases on the part of the parents. Common reasons for refusal were related to the content of the intervention and concerns about girls leaving the ITS to attend sessions; in some cases, an entire ITS opted out of the study.

### Surveys and dataset

A household survey and a girls’ survey were administered in Arabic via an open data kit platform [28]. The household survey included a household roster that captured socio-demographic characteristics of household members and an asset index that was taken from the UNHCR Vulnerability Assessment Toolkit [29]. The household survey was administered by a CW in the household residence. In most cases, the survey questionnaire was completed by the mother.

The girls’ survey captured measures of the main outcomes of the intervention, including: school status and experiences; attitudes toward education, marriage and gender roles; and experiences, knowledge and attitudes around menstruation. Girls aged 15–17 and all girls who were married, regardless of age, were asked about knowledge of contraception and STIs. Due to sociocultural taboos around asking unmarried adolescent girls in the study population about topics related to sex, girls younger than 15 to 17 were not administered questions on contraception or STIs. Female graduate students from the study team’s university administered the questionnaire to girls at a local community center or in the ITS where they lived.

While we planned to administer the household survey immediately following parental consent, some parents were unavailable to complete the survey at the time of consent. When data collection was halted, some of these households had still not been surveyed. We therefore have surveys from 429 girls in 310 households, but 184 girls in 140 households are missing household-level data. Finally, recruitment of married adolescents proved very challenging; only 11 consented to be surveyed. We excluded these girls from the dataset, since their experiences with SRH are likely to be very different from girls who were unmarried. Our analytic sample consists of 418 unmarried girls in 305 households.

### Outcomes

The study outcome variables capture girls’ knowledge of puberty and sexual and reproductive health. The first outcome measures girls’ accurate knowledge of puberty, constructed as an additive index of the following seven items: i) Changes in the body during puberty happen because of hormones; ii) Girls’ and boys’ bodies begin to change around the age of 10; iii) Women stop menstruating after the age of about 40–50; iv) Menstruation in girls and women is a normal process and not a sickness; v) Once a girl begins to menstruate, she is physically able to get pregnant; vi) Pregnant women menstruate; vii) A girl should not bathe during her period. The last item is related to a common misconception in the study community that women should not bathe while menstruating. Answer choices were “yes,” “no” and “don’t know.” All items were recoded such that the correct answer equaled 1 and other answers 0, then the seven items were summed to obtain the total knowledge score.

For girls aged 15–17, we examine three other SRH outcomes. Participants were asked whether they recognized the following contraceptive methods: oral contraceptive pill, intra-uterine device, male condom, injectables, implants, emergency contraceptive pill, lactational amenorrhea (breastfeeding), withdrawal and periodic abstinence/rhythm method. The binary outcome “knowledge of at least one LARC method” captures whether the girl reported knowing of at least one of the following three long-acting reversible contraceptive (LARC) methods: the intra-uterine device, injectable or implant.

Our remaining two outcomes consist of binary measures of whether the participant 1) had heard of HIV/AIDS and 2) had heard of any sexually transmitted infection (STI) other than HIV/AIDs. To assess knowledge of HIV transmission, we asked participants whether the following mechanisms were modes of transmission: kissing, sexual relations, contaminated blood (transfusion), sharing a needle, insect bite, sharing food with an infected person and from mother to child during pregnancy. Answer choices were “yes,” “no” and “don’t know.” All items were recoded such that the correct answer equaled 1 and other answers 0. Although the survey included questions to assess knowledge of STI transmission, so few participants had heard of STIs that these questions were not analyzable.

The measures for puberty knowledge and HIV transmission were developed by the study team, rather than using validated scales, based on our prior work with the study population and pre-testing of the survey with unmarried and married girls of different ages and literacy levels. Pre-testing revealed that knowledge of SRH-related terms, such as “STI,” was very low among the study population, which has low literacy levels overall. Revisions were made to the questionnaire to facilitate participant understanding of key terms. The team also thought that it was important for knowledge-related survey items to capture common misconceptions among the study population, such as the issue of bathing while menstruating, as these would be addressed in the intervention. We therefore chose to focus on more basic SRH knowledge items (e.g. knowing what an STI is rather than identifying symptoms) and on contextualized measures rather than use of existing scales.

### Independent variables

Our key independent variables of interest capture mother-daughter communication around puberty and girls’ schooling experiences. Mother-daughter communication is a binary indicator equal to 1 if the girl reported that she had ever spoken to her mother about puberty or menstruation prior to getting her first period, and equal 0 if she had not. We use this communication variable in all of our analyses, including those of SRH outcomes, as a proxy for the openness of the mother in talking to her daughter about SRH issues.

Schooling experiences are captured for all girls in two variables: 1) a binary indicator of whether the girl was enrolled in school at the time of the survey; and 2) a variable indicating the highest grade level she ever reached in school, regardless of current enrollment status (coded as 3^rd^ grade or less, 4^th^-5^th^ grade, 6^th^-7^th^ grade, and 8^th^-11^th^ grade). The latter variable is important because reproduction is covered in 6^th^ and 7^th^ grade biology in the Lebanese school curriculum [14] and HIV/AIDS in 8^th^ grade [30]. Highest grade reached is therefore a proxy for likely exposure to any SRH content in school, both for girls who remain in school and those who have dropped out. For analyses restricted to currently in-school girls, we include an additional covariate capturing whether the girl reported learning about menstruation in school (equal to 1 if “yes”). This question was only asked to currently in-school girls in the survey due to concerns about recall bias for those who had left school.

Other covariates included in the analysis are informed by the conceptual model in **Fig 1**. At the individual level, these include girl’s age to account for maturation and the possibility that girls acquire SRH knowledge through other mechanisms, such as peer networks, as they age. We also include variables that capture other, potentially confounding sources of knowledge around SRH: 1) media access, constructed as an additive index ranging from 1–4 indicating whether girls had access to TV, a computer, a mobile phone and/or social media; 2) a binary indicator of whether the girl participated in the 2018 *Amenah* pilot, which covered material related to menstruation (although not SRH); and 3) a binary indicator of whether the girl reported learning about girls’ health or puberty in another activity. The latter variable was included because many NGOs provide ad-hoc health and awareness sessions in the study context, and girls may have received SRH content if they participated in these activities.

At the household level, we examine residence (coded as ITS vs. community setting) and wealth. The wealth variable was constructed using factor analysis of the 29-item UNHCR asset index [29]; households were categorized as being in the upper or lower half of the wealth distribution. We also examine mother’s education, coded as primary school or less vs. preparatory (8^th^ grade) or more, because parental education is correlated with girls’ SRH knowledge in some contexts [22].

### Analysis

We first present summary statistics for our independent variables for the full sample of girls (N = 418), all girls who had reached menarche (N = 261), in-school girls who had reached menarche (N = 148), and girls aged 15–17 (N = 106). We also descriptively analyzed outcomes related to which contraceptive methods girls knew and their knowledge of HIV transmission mechanisms, presenting graphically the percentage of girls who knew of the method or correctly identified the transmission mechanism, respectively.

We conducted logistic regression analysis for all outcomes analyzed in a multivariate framework, except for the knowledge of puberty scale, for which we conducted ordinary least squares regression. It is important to note that while the knowledge of puberty scale was collected for all girls who participated in the survey, one of our key covariates of interest–mother-daughter communication prior to reaching menarche–is by definition only applicable to girls who had reached menarche. To maintain consistency, we therefore restrict all analyses related to the puberty outcome to the sample of girls who had reached menarche. We additionally analyzed puberty knowledge among currently in-school participants who had reached menarche to better understand the association between having received information on menstruation in school and puberty knowledge. All 15-17-year-old participants had reached menarche, so this was not a concern for the other SRH outcomes.

Models were constructed in a step-wise fashion and only independent variables that were significant at the p<0.2 level in bivariate analysis were included in the multivariate models. The exceptions are the girl’s age, which was included in all multivariate models to account for maturation, as well as our key independent variables of interest: current school enrollment and prior maternal puberty-related communication. Household- and mother-level independent variables were not included in the bivariate or multivariate analyses due to the large amount of missing data. Although we cannot assess this empirically because all socioeconomic variables were collected in the household survey, we expect that girls for whom household data were missing were on average of higher socioeconomic status because data collection commenced in camps (which tend to have lower SES) and then moved to community settings. More girls in community settings were therefore missing household data. Standard errors in all models are clustered at household level due to the presence of sisters in the data. All descriptive and multivariate analyses were conducted using STATA 16 [31].

## Results

### Characteristics of participants

**Table 1** presents descriptive analysis of our independent variables for the full sample of girls, all girls who had reached menarche, in-school girls who had reached menarche, and girls aged 15–17.

**Table 1:** Summary statistics for independent variables, full sample, girls who had reached menarche, in-school girls who had reached menarche, and girls aged 15–17.

Characteristic	Full sample (N = 418)	Participants who had reached menarche (N = 261)	In-school participants who had reached menarche (N = 148)	Participants aged 15–17 (N = 106)
**Key independent variables of interest**				
Discussed menstruation with mother prior to menarche (%)	N/A	32.6 (85)	33.8 (50)	35.8 (38)
Currently enrolled in school (%)	65.6 (274)	56.7 (148)	N/A	36.8 (39)
Highest grade attained ^a^ (%)				
• 3^rd^ grade or lower	23.5 (98)	17.2 (45)	6.1 (9)	16.0 (17)
• 4^th^ - 5^th^ grade	36.2 (151)	30.7 (80)	29.1 (43)	25.5 (27)
• 6^th^ - 7^th^ grade	29.0 (121)	34.1 (89)	39.9 (59)	29.2 (31)
• 8^th^ - 11^th^ grade	11.3 (47)	18.0 (47)	25.0 (37)	29.2 (31)
Learned about menstruation in school	N/A	N/A	26.5 (39)	N/A
**Individual characteristics**				
Age in years (mean)	13.4	14.3	13.8	15.8
Access to media devices (mean number)	2.5	2.7	2.8	2.7
Participation in pilot study (%)	16.5 (69)	23.0 (60)	32.4 (48)	26.4 (28)
Participation in other health or puberty programs (%)	17.7 (74)	20.7 (54)	19.6 (29)	23.6 (25)
**Household characteristics**				
Residence (%)				
• Informal tented settlement (camp)	48.6 (203)	46.0 (120)	29.7 (44)	48.1 (51)
• Community setting (non-camp)	14.6 (61)	16.1 (42)	21.6 (32)	12.3 (13)
• Missing data Wealth (%)	36.8 (154)	37.9 (99)	48.6 (72)	39.6 (42)
• Lower half	25.6 (107)	23.0 (60)	15.5 (23)	24.5 (26)
• Upper half	30.4 (127)	31.8 (83)	29.1 (43)	31.1 (33)
• Missing data	44.0 (184)	45.2 (118)	55.4 (82)	44.3 (47)
**Mother characteristics**				
Mother’s highest education level (%)				
• Primary school or lower	37.1 (155)	34.1 (89)	22.3 (33)	37.7 (40)
• Preparatory school or above	17.9 (75)	20.3 (53)	22.3 (33)	17.0 (18)
• Missing data	45.0 (188)	45.6 (119)	55.4 (82)	45.3 (48)

^a^ Data missing for one out-of-school participant.

Only about a third of participants who had reached menarche had discussed puberty or menstruation with their mothers prior to getting their first period. Two-thirds of participants were enrolled in school at the time of the survey, as compared to 56.7% of girls who had reached menarche and only 36.8% of girls aged 15–17. Highest grade reached varied considerably among the sample, a point we return to below. Among girls who were currently in school at the time of the survey, only 26.5% had learned about menstruation in school.

As expected, girls who had reached menarche were on average older (14.3 years) than the overall sample (13.4 years). They were somewhat more likely to have participated either in the Amenah pilot or another girls’ health- or puberty-related activity, as were girls in school and those aged 15–17. Access to media was 2.5–2.8 devices out of the four for all sample groups. About half of participants lived in an ITS, although as noted in the methods data were missing on this variable for nearly 40% of girls. Finally, mothers’ education levels were low overall; among those with valid data, the majority of mothers had attended only primary school or less.

Since grade reached in school may determine girls’ exposure to any SRH-related curriculum content, **Table 2** delves further into the issue of grade-for-age variability among the study participants. In addition to sharp declines in enrollment rates observed from age 15, many in-school girls were behind in terms of expected grade-for-age. For example, nearly a third of in-school 13-year-olds were in 4^th^ or 5^th^ grade. Given that dropout rates increase considerably around age 15 in this population, these girls may never reach beyond primary school.

**Table 2:** Participants’ current school status and grade by age.

		Current grade among those in school (N)				
Age	Not in school (%)	3rd grade or less	4th-5th grade	6th-7th grade	8th-11th grade	Total
11	16 (23.2)	20	29	4	0	69
12	10 (14.3)	11	34	15	0	70
13	26 (26.5)	5	31	36	0	98
14	25 (33.3)	3	9	25	13	75
15	25 (53.2)	0	3	9	10	47
16	19 (61.3)	0	0	2	10	31
17	23 (82.1)	0	1	0	4	28
**Total**	**144 (34.5)**	**39**	**107**	**91**	**37**	**418**

### Knowledge of puberty

Among all participants who had reached menarche, the mean number of correct responses on the puberty knowledge scale was 3.7 out of 7 (**Table 3**). The mean score among in-school participants was slightly lower (3.4).

**Table 3:** Summary statistics for knowledge outcomes.

Participants who had experienced menarche (N = 261)	
Mean puberty knowledge score (SD)	3.7 (1.5)
**In-school participants who had experienced menarche (N = 148)**	
Mean puberty knowledge score (SD)	3.4 (1.5)
**Participants aged 15–17 years (N = 106)**	
Identify at least one LARC method (%)	64.2
Knowledge of HIV (%)	56.6
Knowledge of STIs (%)	23.6

Older age (0.35; 95% CI 0.24–0.45; p<0.001) and participation in a girls’ health program (0.49; 95% CI 0.06–0.91; p<0.05) were strongly associated with higher puberty knowledge among all girls who had reached menarche in bivariate analyses (**Table 4**).

**Table 4:** Bivariate analysis for knowledge outcomes of interest.

Variable	Puberty knowledge score^†^^¥^ (95% CI)	Knowledge of HIV^‡^^π^ (95% CI)	Knowledge of STIs^‡^^π^ (95% CI)	Knowledge of LARC method^‡^^π^ (95% CI)
**Age**	0.35 (0.24–0.45)***	2.03 (1.25–3.30)**	1.21 (0.69–2.11)	2.08 (1.23–3.53)**
**Currently enrolled in school**	-0.57 (-0.94 - -0.19)**	2.32 (1.02–5.29)*	1.85 (0.74–4.63)^□^	0.59 (0.26–1.36)^□^
**Highest grade reached (ref: < = 3**^**rd**^ **grade)**	**	**	^□^	
• **4**^**th**^**-5**^**th**^ **grade**	-0.37 (-0.91–0.17)	1.92 (0.51–7.18)	0.93 (0.20–4.23)	0.62 (0.16–2.41)
• **6**^**th**^**-7**^**th**^ **grade**	-0.45 (-0.99–0.09)	1.73 (0.49–6.07)	0.35 (0.07–1.76)	0.43 (0.11–1.59)
• **8**^**th**^**-11**^**th**^ **grade**	0.41 (-0.22–1.04)	72 (7.51–690.53)***	2.05 (0.54–7.84)	0.49 (0.13–1.86)
**Access to media devices**	0.11 (-0.09–0.31)	1.48 (0.98–2.26)^□^	1.64 (0.93–2.86)^□^	0.87 (0.58–1.29)
**Prior maternal puberty communication**	0.05 (-0.37–0.47)	2.60 (1.09–6.22)*	1.95 (0.78–4.91)^□^	1.62 (0.68–3.85)
**Participation in pilot intervention**	-0.11(-0.57–0.35)	1.26 (0.52–3.05)	1.11 (0.41–3.04)	1.01 (0.41–2.46)
**Participation in other girls’ health activity**	0.49 (0.06–0.91)*	1.20 (0.48–3.01)	3.76 (1.41–10.02)**	1.59 (0.59–4.32)

^†^ Presented as correlation coefficient based on ordinary least squares regression.

^‡^ Presented as Odds Ratio based on logistic regression.

^¥^ Outcome measured among participants who reached menarche (N = 261).

^π^ Outcome measured among participants aged 15–17 years (N = 106).

^□^ p≤0.2.

*p<0.05.

**p<0.01.

***p<0.001.

In contrast, current school enrollment was significantly associated with lower puberty knowledge (-0.57; 95% CI -0.94- -0.19; p<0.01), likely a function of age. Highest grade reached was jointly significant but without a consistent pattern across grade levels. When restricting the bivariate analysis to the sub-sample of girls who reached menarche and were currently enrolled in school (N = 148), as seen in **Table 5**, learning about menstruation in school (0.56; 95% CI 0.04–1.08; p<0.05) was found to significantly correlate with a higher puberty knowledge score.

**Table 5:** Bivariate analysis for puberty knowledge score among girls enrolled in school who had reached menarche (N = 148).

Variable	Puberty knowledge score^†^ (95% CI)
**Age**	0.33 (0.17–0.49)***
**Learned about menstruation in school**	0.56 (0.04–1.08)*
**Highest grade reached (ref: < = 3**^**rd**^ **grade)**	***
4^th^-5^th^ grade	-0.86 (-1.54- -0.18)*
6^th^-7^th^ grade	-1.00 (-1.66- -0.34)**
8^th^-11^th^ grade	0.22 (-0.51–0.94)
**Access to media devices**	0.09 (-0.19–0.38)
**Prior maternal puberty communication**	0.32 (-0.21–0.86)
**Participation in pilot intervention**	0.08 (-0.47–0.62)
**Participation in other girls’ health activity**	0.44 (-0.16–1.04) ^□^

^†^ Presented as correlation coefficient based on ordinary least squares regression.

^□^ p≤0.2.

* p<0.05.

**p<0.01.

***p<0.001.

In the multivariate analysis (**Table 6**), both age (0.27; 95% CI 0.12–0.41; p<0.001) and participation in a girls’ health program other than the pilot intervention (0.41; 95% CI 0.02–0.81; p<0.05) retained significance for the sample of all girls who had reached menarche. Among in-school girls who had reached menarche, age (0.20; 95% CI 0.00–0.41; p<0.05) retained a significant association with a higher knowledge score in the multivariate analysis and certain grade levels with a lower knowledge score (**Table 7**). The unexpected pattern seen for highest grade reached may be a result of the fact that very few in-school girls were in 3^rd^ grade or lower (N = 9).

**Table 6:** Multivariate analysis of knowledge outcomes of interest.

Variable	Puberty knowledge score^†^^¥^ (95% CI)	Knowledge of HIV^‡^^π^ (95% CI)	Knowledge of STIs^‡^^π^ (95% CI)	Knowledge of LARC method^‡^^π^ (95% CI)
**Age**	0.27 (0.12–0.41)***	1.97 (1.07–3.65)*	1.10 (0.52–2.32)	1.94 (1.12–3.34)*
**Current school enrollment**	-0.28 (-0.70–0.14)	0.45 (0.11–1.86)	1.83 (0.37–9.05)	0.66 (0.26–1.67)
**Highest grade reached (ref: < = 3**^**rd**^ **grade)**				
**4**^**th**^**-5**^**th**^ **grade**	-0.20 (-0.74–0.35)	2.20 (0.57–8.58)	0.81 (0.15–4.47)	--
**6**^**th**^**-7**^**th**^ **grade**	-0.33 (-0.90–0.23)	2.58 (0.68–9.83)	0.12 (0.01–1.41)	--
**8**^**th**^**-11**^**th**^ **grade**	0.33 (-0.43–1.09)	151.38 (9.38–2442.92)***	0.78 (0.10–6.20)	--
**Access to media devices**	--	1.00 (0.53–1.90)	1.60 (0.75–3.43)	--
**Prior maternal puberty communication**	-0.16 (-0.56–0.25)	2.12 (0.70–6.48)	1.16 (0.44–3.07)	1.67 (0.65–4.33)
**Participation in other girls’ health program**	0.41 (0.02–0.81)*	--	4.21 (1.45–12.28)**	--

^†^ Presented as correlation coefficient based on ordinary least squares regression.

^‡^ Presented as Odds Ratio based on logistic regression.

^¥^ Outcome measured among participants who reached menarche (N = 261).

^π^ Outcome measured among participants aged 15–17 years (N = 106).

* p<0.05.

**p<0.01.

***p<0.001.

**Table 7:** Multivariate analysis for puberty knowledge score among girls enrolled in school who had reached menarche (N = 148).

Variable	Puberty knowledge score^†^ (95% CI)
**Age**	0.20 (0.00–0.41)*
**Learned about menstruation in school**	0.12 (-0.48–0.72)
**Highest grade reached (ref: < = 3**^**rd**^ **grade)**	
4^th^-5^th^ grade	-0.81 (-1.52 –-0.90)*
6^th^-7^th^ grade	-1.17 (-1.89 –-0.44)**
8^th^-11^th^ grade	-0.23 (-1.34–0.87)
**Prior maternal puberty communication**	0.08 (-0.44–0.61)
**Participation in other girls’ health activity**	0.33 (-0.19–0.85)

^†^ Presented as correlation coefficient based on ordinary least squares regression.

^□^ p≤0.2.

* p<0.05.

**p<0.01.

***p<0.001.

### Knowledge of LARC methods

**Fig 2:**
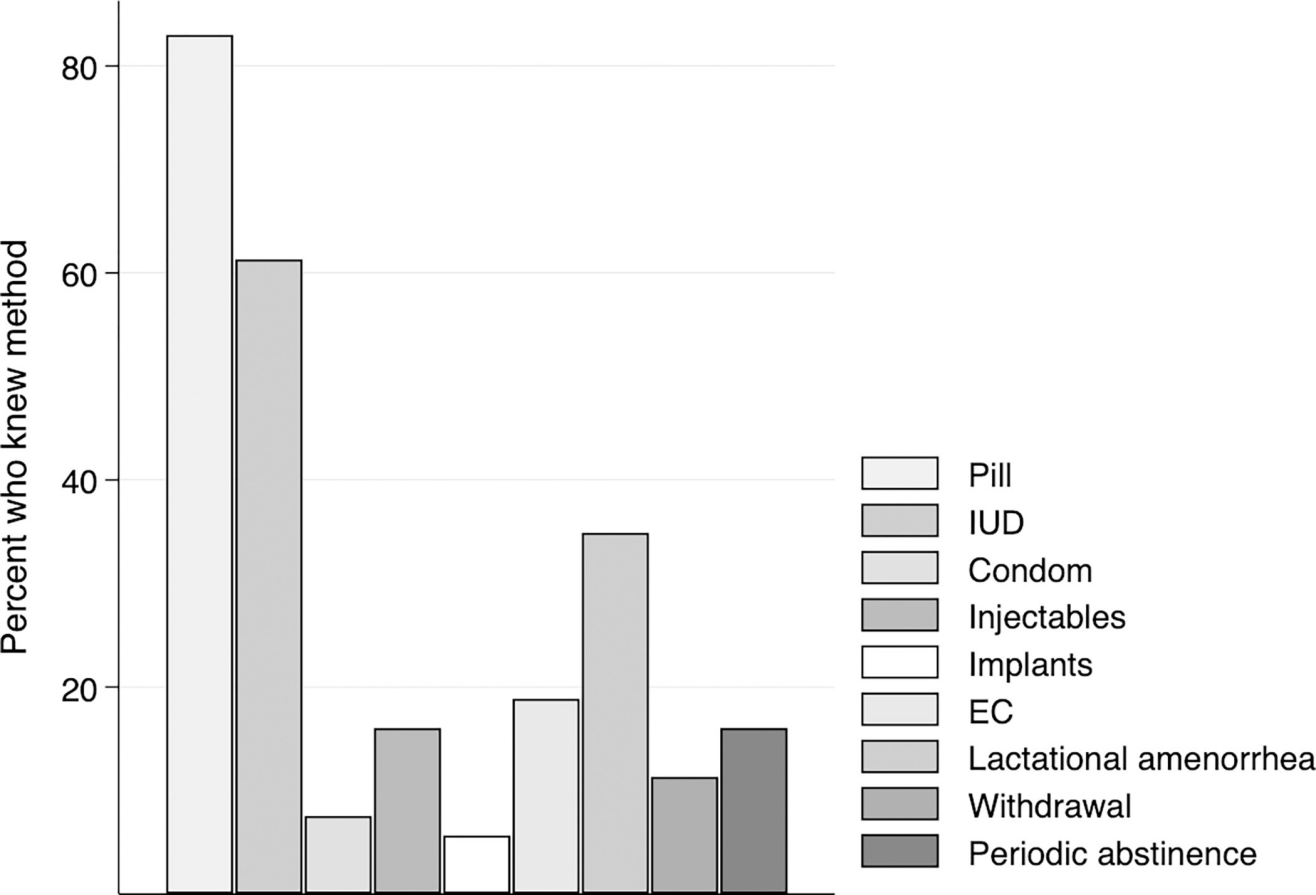
Knowledge of contraceptive methods among 15-17-year-old participants (N = 106). Note: IUD—Intra-uterine device; EC—emergency contraception.

As shown in **Table 3**, 64.2% of 15-17-year-old girls were able to identify at least one LARC method. Oral contraceptive pills were the most common contraceptive method recognized by study participants (83.0%), followed by intrauterine devices (61.3%) (**Fig 2**). Less than 10% of girls identified condoms or implantable devices. In bivariate models (**Table 4**), only age was found to be associated with identifying at least one LARC method (OR 2.08; 95% CI 1.23–3.53; p<0.01). This association was similarly observed in multivariate analysis (**Table 6;** OR 1.94; 95% CI 1.12–3.34; p<0.05).

### Knowledge of STIs including HIV/AIDS

Only 23.6% of 15-17-year-old girls recognized the term “sexually transmitted infection” (**Table 3**). Having participated in a girls’ health program outside of school was strongly associated with recognition of STIs, both in bivariate (**Table 4;** OR 3.76; 95% CI 1.41–10.02; p<0.01) and multivariate models (**Table 6;** OR 4.21; 95% CI 1.45–12.28; p<0.01). None of the other covariates were found to be associated with this outcome, possibly in part due to the small sample size.

**Fig 3:**
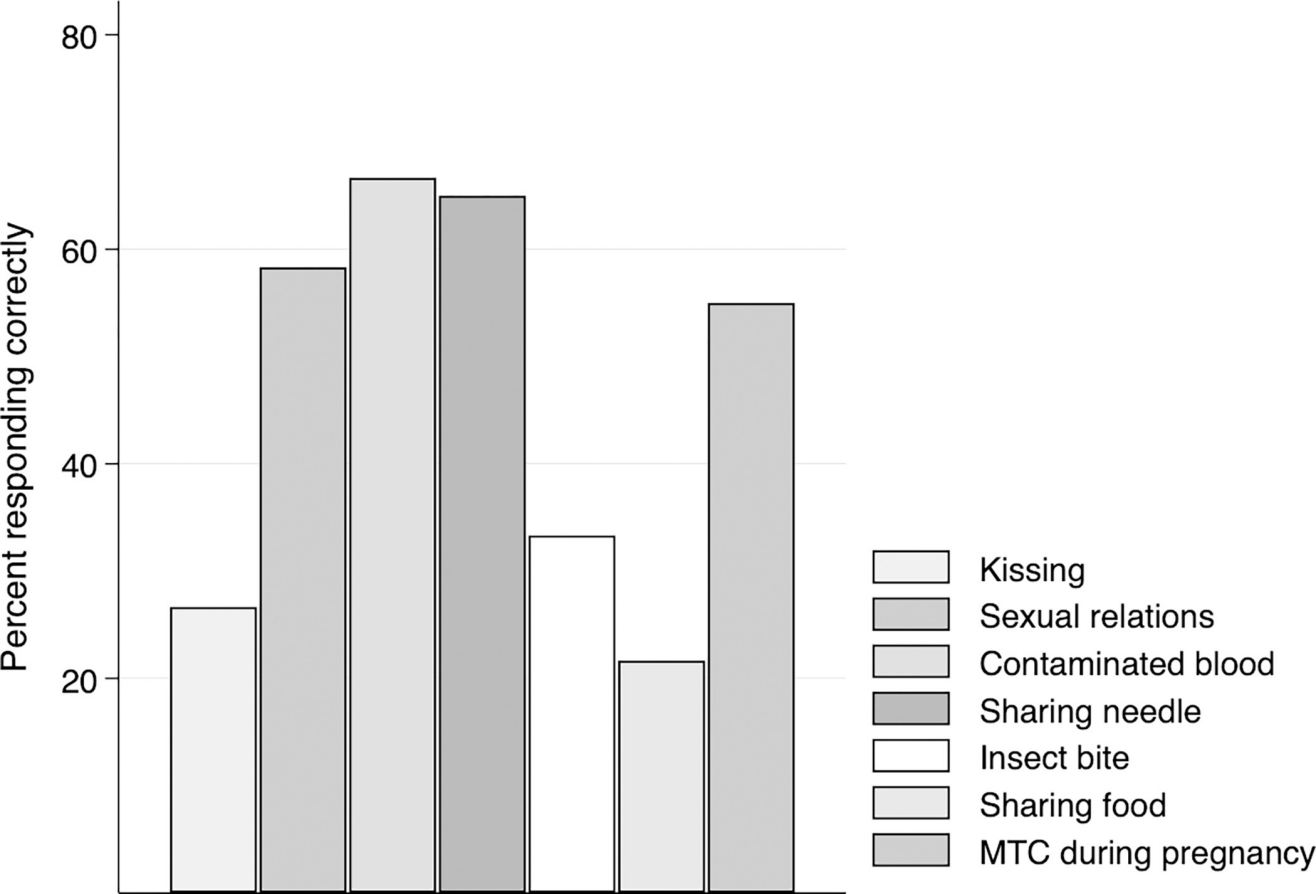
Knowledge of mechanisms of HIV transmission among 15-17-year-old participants who had heard of HIV (N = 60).

By contrast, 56.6% of 15-17-year-olds had heard of HIV/AIDS (**Table 3**). Among participants who recognized the term “HIV/AIDS”, 58.3% identified sexual intercourse, 66.7% contaminated blood transfusion and 65.0% needle-sharing as methods of transmission (**Fig 3**).

In bivariate analysis, age (OR 2.03; 95% CI 1.25–3.30; p<0.01), current school enrollment (OR 2.32; 95% CI 1.02–5.29; p<0.05), and having discussed menstruation with their mother (OR 2.60; 95% CI 1.09–6.22; p<0.05) were significantly associated with HIV knowledge (**Table 4**). In bivariate models, having reached the 8^th^-11^th^ grade almost perfectly correlated with HIV knowledge (OR 72; 95% CI 7.51–690.53; P<0.001), as all but one of this fairly small group of participants (N = 30) demonstrated knowledge of HIV infection. Reaching the 8^th^-11^th^ grade remained significantly correlated (OR 152; 95% CI 12.95–1784.52; p<0.001) in multivariate models (**Table 6**), as did age (OR 1.97; 95% CI 1.07–3.63; p<0.05).

## Discussion

This is the first study to quantitatively examine correlates of SRH knowledge among a vulnerable population of forcibly displaced Syrian adolescent girls in Lebanon. We find that girls generally have low SRH knowledge and many do not receive information on puberty, whether from mothers or school. The fact that age is the most consistent correlate of SRH knowledge suggests that many girls are learning about these topics through informal channels, such as discussions with peers or life experience. This leads to important questions about the accuracy and completeness of the information to which girls have access, particularly given strong social norms against unmarried girls discussing matters related to sex and reproduction [6, 8, 14].

Our results do suggest that schooling, more than maternal communication, may improve SRH knowledge among this population. Although there were few significant results in the multivariate analyses, possibly due to small sample sizes, the bivariate results indicate that learning about puberty in school and being in higher grades are associated with greater knowledge of puberty and HIV, respectively. The association between reaching 8^th^-11^th^ grade and HIV knowledge is well-aligned with what is taught about SRH in Lebanese public schools [14], where reproduction is included in the 6^th^ and 7^th^ grade biology curriculum and HIV/AIDS in 8^th^ grade [30], which suggests that Syrian students are receiving the same content as Lebanese adolescents.

Our findings regarding schooling are particularly important given that adolescent Syrian refugees suffer from among the lowest school enrollment rates in the world [6, 32]. Our study findings thus offer insight into how conflict and displacement may indirectly and longitudinally impact girls’ health and well-being, with implications for adolescents in contexts of forced migration worldwide. Globally, refugee children suffer from low gross enrollment ratios in secondary school (36% compared to a global average of 67%) [33]. Yet, school-based SRH education programs in LMICs are effective at reducing high-risk sexual behavior and improving SRH self-efficacy [34]. Though our results show that ad-hoc health programs outside of school may help fill some of these gaps, facilitating schooling opportunities for refugee girls remains potentially the most critical step in improving their long-term SRH, as school-based initiatives are cost-efficient and can reach girls at scale.

The association between grade reached and knowledge of certain SRH topics is notable in this population, which has considerable age-for-grade heterogeneity. This trend is also observed among diverse populations of adolescents living in LMICs and may be mediated by poverty, gender, and early childhood growth among other factors [35, 36]. Furthermore, early sexual debut, early marriage and adolescent pregnancy disproportionately predict school dropout among low-income female adolescents in multiple LMICs in Sub-Saharan Africa [37, 38]. It is essential, therefore, that age-appropriate CSE be made available to children and adolescents irrespective of their grade in order to reach those highest risk adolescents. While CSE may be controversial in conservative contexts, including that of Syrians displaced in Lebanon, the importance of universal CSE is further underscored by the association between early marriage and school dropout among girls in this community [9], as girls’ mobility, and therefore access to SRH information and services, becomes significantly constrained following marriage [6, 39].

In addition to SRH curricula exposure, there may other factors related to school attendance that positively impact SRH knowledge and attitudes. The relationship between education and numerous reproductive health outcomes among women, including lower actual and wanted fertility rates, higher age at first marriage, and greater utilization of modern contraceptives, has long been demonstrated in LMICs [40, 41]. The mechanisms by which education influences reproductive health outcomes are myriad and include pragmatic skills such as literacy and numeracy that may facilitate medical decision-making in adulthood, as well as more implicit factors such as shaping identity and promoting self-efficacy [41–43]. We therefore recommend that SRH programs for out-of-school Syrian refugee adolescents include educational content to improve general literacy, as this may bolster self-empowerment and confer additional health benefits.

Our results also suggest that maternal-child SRH communication in this population is not significantly associated with improved SRH knowledge. These observations are supported by qualitative data showing that un- and early-married Syrian refugee girls primarily receive delayed and incomplete SRH information from their mothers [14]. Potential barriers to productive discourse include shame and stigma, lack of information and education among mothers, as fewer than 20% of mothers surveyed completed secondary school, making it unlikely that they received formal SRH education or are even literate. Nevertheless, numerous studies from the region have found that mothers are girls’ primary and preferred source of SRH information [22, 44]. Our findings emphasize the importance of including mothers in SRH educational interventions in this community, focusing not only on providing correct information but also on improving communication, which may translate into safer practices among adolescents [45]. Indeed, the *Amenah* intervention includes a dedicated component for mothers which focuses on destigmatizing SRH and improving maternal-daughter communication around menstruation and other SRH topics [46].

While our study is novel, there are several limitations to consider when interpreting the results. Firstly, the population sampled is not representative of Syrian refugees in Lebanon or in the Beqaa. For instance, nearly half of our sample resided in a tented, non-permanent residence at the time of the survey, compared with 21% of Syrian refugees in Lebanon in 2020 [47]. Secondly, the substantial amount of missing data at the household level, a result of the interruption of the data collection by the COVID-19 pandemic, precluded the inclusion of socioeconomic covariates in the analysis. Our sample size is also small, particularly subgroup analyses of in-school girls and those aged 15–17 (the latter likely due to higher prevalence of marriage among older adolescents), which led to wide confidence intervals on many estimates and the inability to analyze certain outcomes of interest for the older age group. The study also did not include boys, who likely obtain sexual health information and services through distinct channels than do girls. Additionally, the study, which focused on schooling and maternal communication, did not address all of the SRH determinants outlined in our conceptual framework (**Fig 1**). Many of these determinants, such as humanitarian crises, extreme poverty, and early marriage are societal- and community-based drivers of health which are beyond the scope of this current work. Notably, however, the *Amenah* intervention does explicitly address several of these issues, including patriarchal gender norms, drivers of early marriage, access to health services, stigma around girls’ SRH and adolescent disempowerment [46]. Finally, due to gender norms and stigmatization of premarital sex, we were only able to measure knowledge, rather than attitudinal or behavioral outcomes, though studies elsewhere have shown that SRH knowledge in adolescence may be protective against high-risk sexual practices in adulthood [48, 49].

## Conclusion

In conclusion, adolescent Syrian refugee girls are an understudied and vulnerable population at risk of negative SRH outcomes. We show that, among a sample of 418 11-17-year-old Syrian refugee girls displaced in the Beqaa governorate of Lebanon, schooling is a stronger correlate of SRH knowledge than is maternal communication. Lebanese schools should administer age-appropriate CSE to students on the basis of their age, rather than grade, given significant age-for-grade heterogeneity among Syrian refugees. SRH educational programs for out-of-school adolescents should include mothers and incorporate literacy skills. Retention in education may be a significant intervention to support adolescent refugee girls’ long-term SRH and well-being.
